# Molecular Genetic Regulation of *Slc30a8*/ZnT8 Reveals a Positive Association With Glucose Tolerance

**DOI:** 10.1210/me.2015-1227

**Published:** 2015-11-19

**Authors:** Ryan K. Mitchell, Ming Hu, Pauline L. Chabosseau, Matthew C. Cane, Gargi Meur, Elisa A. Bellomo, Raffaella Carzaniga, Lucy M. Collinson, Wen-Hong Li, David J. Hodson, Guy A. Rutter

**Affiliations:** Section of Cell Biology and Functional Genomics (R.K.M., M.H., P.L.C., M.C.C., G.M., E.A.B., D.J.H., G.A.R.), Division of Diabetes, Endocrinology and Metabolism, Imperial Centre for Translational and Experimental Medicine, Imperial College London, London W12 0NN, United Kingdom; Electron Microscopy Unit (R.C., L.M.C.), Francis Crick Institute, Lincoln's Inn Fields, London WC2A 3LY, United Kingdom; and Department of Cell Biology and Biochemistry (W.-H.L.), The University of Texas Southwestern Medical Center, Dallas, Texas 75390

## Abstract

Zinc transporter 8 (ZnT8), encoded by *SLC30A8*, is chiefly expressed within pancreatic islet cells, where it mediates zinc (Zn^2+^) uptake into secretory granules. Although a common nonsynonymous polymorphism (R325W), which lowers activity, is associated with increased type 2 diabetes (T2D) risk, rare inactivating mutations in *SLC30A8* have been reported to protect against T2D. Here, we generate and characterize new mouse models to explore the impact on glucose homeostasis of graded changes in ZnT8 activity in the β-cell. Firstly, *Slc30a8* was deleted highly selectively in these cells using the novel deleter strain, Ins1Cre. The resultant Ins1CreZnT8KO mice displayed significant (*P* < .05) impairments in glucose tolerance at 10 weeks of age vs littermate controls, and glucose-induced increases in circulating insulin were inhibited in vivo. Although insulin release from Ins1CreZnT8KO islets was normal, Zn^2+^ release was severely impaired. Conversely, transgenic ZnT8Tg mice, overexpressing the transporter inducibly in the adult β-cell using an insulin promoter-dependent Tet-On system, showed significant (*P* < .01) improvements in glucose tolerance compared with control animals. Glucose-induced insulin secretion from ZnT8Tg islets was severely impaired, whereas Zn^2+^ release was significantly enhanced. Our findings demonstrate that glucose homeostasis in the mouse improves as β-cell ZnT8 activity increases, and remarkably, these changes track Zn^2+^ rather than insulin release in vitro. Activation of ZnT8 in β-cells might therefore provide the basis of a novel approach to treating T2D.

The regulation of insulin secretion by glucose involves the uptake and metabolism of the sugar by pancreatic β-cells (1), stimulation of mitochondrial oxidative metabolism (2), Ca^2+^ influx (3), and the exocytosis of the hormone from dense core secretory granules (4, 5) where it is stored in a near-crystalline form alongside Zn^2+^ and Ca^2+^ ions (6). Although it is increasingly accepted that impaired insulin secretion underlies the development of type 2 diabetes (T2D) (7), a disease affecting more than 8% of the adult population worldwide (8), the mechanisms involved remain poorly understood (9). Nonetheless, disease risk is strongly influenced by both genetic (10) and environmental (11) factors.

A nonsynonymous variant in the *SLC30A8* gene associated with elevated T2D risk was identified by genome-wide association studies (GWAS) in 2007 (12). Expressed almost exclusively in pancreatic β- and α-cells (13–15), *SLC30A8* encodes a secretory granule-resident zinc transporter, zinc transporter 8 (ZnT8), implicated in the accumulation of zinc within these organelles and thus in insulin storage (16). Given these likely roles, *SLC30A8*/ZnT8 has been mooted as a potentially tractable new target for personalized disease therapy.

Subsequent functional studies on the expressed ZnT8 protein (14, 17) demonstrated that the risk (R325) variant is a less active zinc transporter than the protective (W) form. Consequently, possession of risk alleles seems likely to impair insulin crystallization and storage. Supporting this view, mice inactivated globally (14, 18) or selectively in the β-cell (15, 19) for *Slc30a8* revealed striking abnormalities in the formation of dense cores within insulin granules. Surprisingly, however, measurements of insulin release from isolated islets from *Slc30a8* null mice revealed either no change (18) or improved (14, 19) glucose-stimulated insulin secretion from isolated islets or the perfused pancreas, and unchanged insulin content. Despite this, glucose homeostasis and circulating insulin levels were both lowered in ZnT8 null animals. Providing a possible explanation for this conundrum, Tamaki et al (19) demonstrated that the enhanced release of Zn^2+^ ions alongside insulin in W-variant carriers suppresses insulin clearance (and presumably nonproductive insulin signaling) by the liver, favoring insulin action on this, as well as other tissues (notably, adipocytes and skeletal muscle). An observed increase in C-peptide to insulin ratio in human R-carriers supported this model, because the mature hormone, but not proinsulin, is expected to be cleared by the liver. Moreover, *Slc30a8* elimination from the mouse has no effect on insulin processing (14, 18), arguing against a β-cell-autonomous action of the variant on the release of mature vs partially processed forms. Together, the above findings have stimulated the search for activators of the transporter which, by favoring Zn^2+^ accumulation by β-cell secretory granules, may eventually prove useful in the clinic.

However, and challenging the above view, a recent study based largely on Swedish, Finnish, and other Northern European populations, but also including individuals from elsewhere, identified rare (<0.1% of the population) nonsense (truncating) or missense mutations in the *SLC30A8* gene. Unexpectedly, the carrier population showed an approximately 3-fold enrichment for healthy individuals vs those with T2D, implying a protective role for the mutant transporter. Although only a small number of carriers was involved (345 in total of ∼150 000 subjects sequenced) a range of structurally distinct variants was found in cohorts with differing ancestry, providing evidence that the *SLC30A8* mutations, rather than other polymorphisms in the same linkage disequilibrium block, were likely to explain the changes in disease risk.

The above findings are nonetheless difficult to reconcile with the observed increase in T2D risk in carriers of the common risk alleles. Although an activating effect of the identified mutants on the remaining allele cannot be excluded absolutely, an alternative explanation (20) is that a complex interplay between insulin storage and Zn^2+^ release by β-cells, and downstream effects on target tissues including the liver, results in a bimodal (bell-shaped) dependence of T2D risk on ZnT8 activity. Thus, modest decreases in β-cell ZnT8 activity, as observed in carriers of the common risk (R) variants, may act chiefly by lowering β-cell Zn^2+^ secretion, thus enhancing insulin clearance by the liver. On the other hand, a more substantial lowering of ZnT8 activity, engendered by rare loss-of-function alleles, may lead to a more dramatic increase in insulin release from the pancreas, an effect outweighing impaired Zn^2+^ release and altered insulin clearance.

The impact of deleting ZnT8 from the β-cell in mice has also been the subject of some debate. Thus, one recent study (21) reported that global knockout on a pure C57BL6 background exerted no effects on glucose tolerance, in contrast to findings on more mixed backgrounds (14, 18). Moreover, several previously reported β-cell-selective deletion models are complicated by deletion in other tissues, including the brain, when *Cre* deleter strains (notably, RIP2Cre and Pdx1), with activity in these tissues (22), are used. Correspondingly, RIP2Cre:ZnT8 mice gain more weight vs controls on a high fat diet than observed with globally deleted animals (23). The latter findings argue for a role for ZnT8 in a small number of neuronal cells in which the *Pdx1* or *Ins2* promoter may be at least transiently active during development or at later stages. On the other hand, the mouse insulin promoter 1 *Cre* (MIPCre) used in Ref. 19 may also be affected by the coexpression of GH encoded by the cDNA included in this transgene (24).

Our first goal here was therefore to explore the impact of deleting ZnT8 more specifically in the β-cell, and on a pure C57BL6 background, using a new deleter strain in which the *Ins1* promoter, which is inactive in brain and other tissues (22), drives expression of Cre after introduction into the endogenous locus (“knock-in”) (25, 26). Importantly, Ins1Cre mice do not express the GH minigene, unlike both RIP2Cre (24) and MIPCre mice (27), and mice bearing the transgene alone display no abnormalities in glucose tolerance (22) (Rutter, G.A., unpublished results).

Up to now, there have been no attempts to examine the effect of overexpressing ZnT8 selectively in the β-cell, thus mimicking one of the likely actions of agents capable of stimulating the activity of the transporter. Our second goal here was therefore to generate a series of transgenic mouse lines in which ZnT8 expression is under the control of rat insulin promoter Tet-On system (28).

We demonstrate that highly selective deletion of ZnT8 in the β-cell leads to dense core granule misformation and glucose intolerance. By contrast, overexpression of the transporter in the β-cell in adults leads to improved glucose tolerance but reduced insulin secretion, whereas Zn^2+^ release is markedly enhanced. A positive relationship thus pertains between β-cell ZnT8 expression (and Zn^2+^ secretion), and glucose tolerance. If reflective of human physiology, these results lend weight to the view that ZnT8 activation might prove beneficial in the context of T2D.

## Results

### Impaired glucose tolerance and insulin secretion in Ins1Cre:ZnT8^fl/fl^ mice

**Figure 1.**
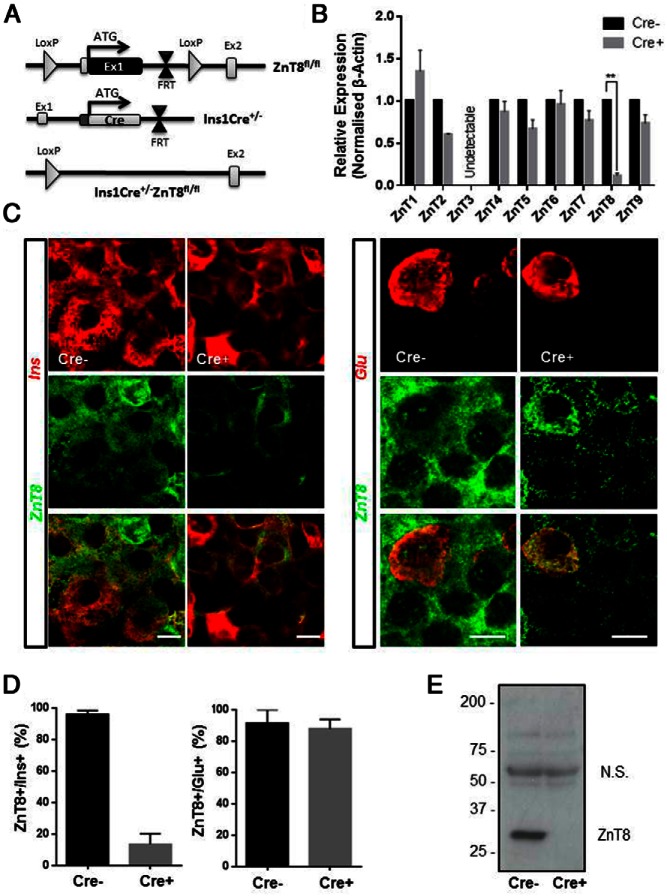
Ins1Cre-mediated deletion of ZnT8 in pancreatic β-cells. Mice carrying a LoxP site together with a flippase recognition target-flanked neomycin selection cassette within intron 1, and a single distal LoxP site within the upstream exon 1 containing the translational start codon, were bred with the Ins1Cre deleter strain, leading to the removal of exon 1 of *Slc30A8*/ZnT8 (A). Ins1Cre-mediated deletion of ZnT8 resulted in an approximate 80%–90% reduction in *Slc30a8* expression (B) (**, *P* < .001 vs 3 Cre^−^, 4 Cre^+^), with no significant changes in the expression of other ZnT family members (*P* > .05 by two-way ANOVA). Gene expression was normalized to β-actin, and fold change gene expression was determined using 2^−ΔΔCT^. B, ZnT8 protein expression is reduced in islets from Cre^+^ animals, as shown by immunofluorescence staining, which demonstrates deletion specifically in β-cells (n = 90) but not α-cells (n = 24) (C and D). Deletion of ZnT8 revealed by Western (immuno)blotting of isolated islets from Ins1Cre^+/−^:ZnT8^fl/fl^ mice and controls (E). Values represent mean ± SEM. Scale bar in C, 12.5 μm.

β-Cell-selective deletion of ZnT8 with a variety of Cre deleter strains (eg, RIP2 [15] and MIP [19]) display varying degrees of recombination at extrapancreatic sites, due to ectopic expression of Cre. By contrast, Ins1Cre knockin mice display no detectable expression of the recombinase in the brain, only very minor recombination in other islet cells (<3% of α-cells in utero), but more than 94% recombination in β-cells (25, 29). We therefore used this model to inactivate ZnT8 selectively in β-cells (Figure 1A). Confirming efficient deletion of the endogenous ZnT8 alleles in the β-cell with Ins1Cre, islets from Ins1Cre^+/−^:ZnT8^fl/fl^ (Cre^+^) mice showed more than 80% reduction in *ZnT8* mRNA levels (**, *P* < .01; two-way ANOVA; Cre^−^ vs Cre^+^; n = 3 and 4, respectively) compared with litter mate controls (Ins1 Cre^−/−^:ZnT8^fl/fl^; Cre^−^), with no changes in the expression of other ZnT family members (Figure 1B). Loss of ZnT8 immunoreactivity was seen specifically in the β-cell, and not the α-cell, compartment, as demonstrated using immunohistochemical analysis of isolated islets, staining for insulin and glucagon, respectively (Figure 1C), and counting the number of ZnT8 positive cells colocalized with insulin and glucagon (Figure 1D). A decrease in overall immunoreactivity of more than 90% for monomeric ZnT8 was shown using Western (immuno)blotting analysis (Figure 1E) compatible with an islet composition of approximately 70%–80% β-cells (30) and levels of ZnT8 expression in α-cells about 50% of those in β-cells (Figure 1C and Ref. 14) .

**Figure 2.**
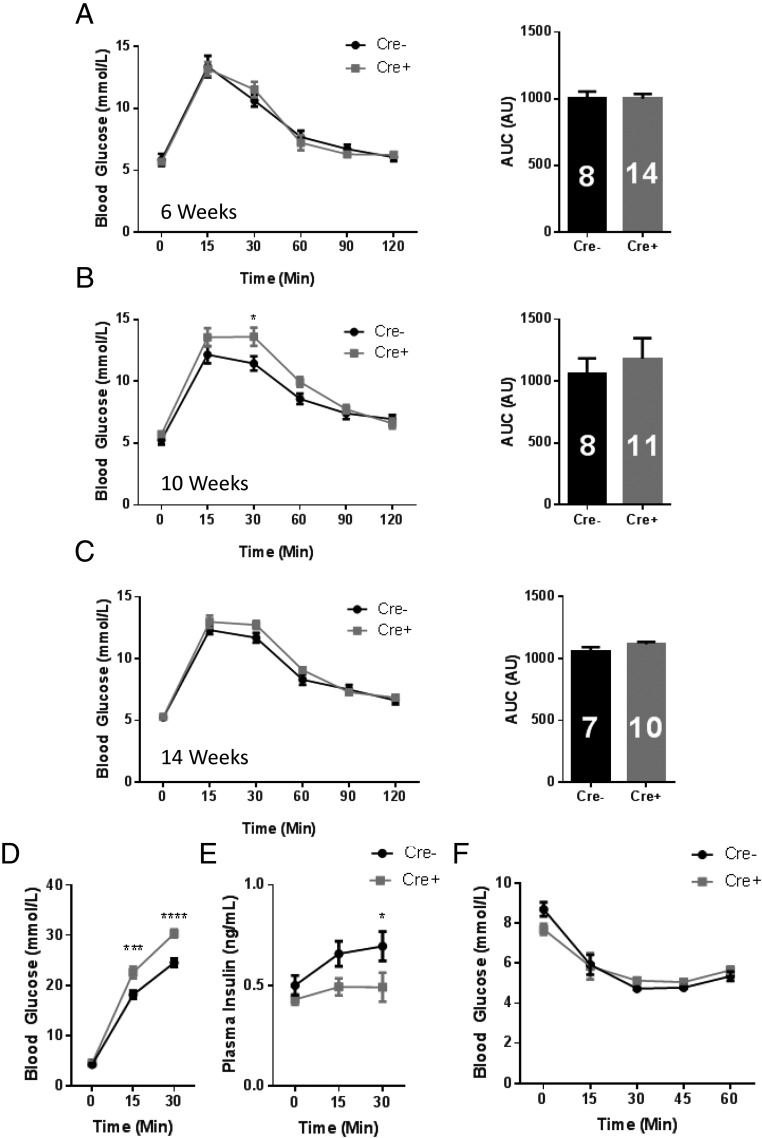
In vivo assessment of glucose homeostasis in Ins1CreZnT8KO mice. Intraperitoneal glucose tolerance test (IPGTT) and AUC of 6-week-old (A), 10-week-old (*, *P* < .05, 30-min time point) (B), and 14-week-old (C) male Ins1Cre^+/−^ZnT8^fl/fl^ (Cre^+^) and littermate control (Ins1CreZnT8^−/−^ZnT8^fl/fl^, Cre^−^) mice. Animals were injected with 1-g/kg bodyweight glucose and blood glucose measured at time point 0, 15, 30, 60, 90, and 120 minutes after glucose injection (n = 7–14 animals). Glucose (*** *P* < .01, 15-min time point; ****, *P* < .001, 30-min time point) (D) and insulin (*, *P* < .05, 30-min time point) (E) responses of 10-week-old male Ins1CreZnT8 mice after 3-g/kg bodyweight glucose injection (n = 13–14 animals per genotype). F, Insulin tolerance test of 10-week-old Ins1CreZnT8 and littermate control male mice. Animals were injected with 0.75-U/kg bodyweight insulin and blood glucose measured as per IPGTT. Numbers in solid bars in the histograms indicate the number of animals studied. Values are mean ± SEM.

Maintained on a regular chow diet, male Ins1Cre^+/−^:ZnT8^fl/fl^ mice displayed normal glucose tolerance at 6 weeks of age (not significant [ns]; repeated measures two-way ANOVA; n = 8 Cre^−^ and n = 14 Cre^+^, respectively) (Figure 2A) but impaired responses to the sugar by 10 weeks (11.5 ± 0.59 vs 13.6 ± 0.74 mmol/L; Cre^−^ vs Cre^+^; *P* < .05; 30-min time point; repeated measures two-way ANOVA; n = 8 and 11, respectively) (Figure 2B). These changes gradually resolved with age (ns; repeated measures two-way ANOVA; n = 7 Cre^−^ and n = 10 Cre^+^) (Figure 2C). Female knockout (KO) mice showed no evident abnormalities at either age (Supplemental Figure 1, A–F). Ins1Cre^+/−^:ZnT8^fl/fl^ (aged 10 wk) showed significantly higher glucose responses (*P* < .001 15- and 30-min time point, repeated measures two-way ANOVA, n = 9 Cre^−^ and n = 12 Cre^+^) (Figure 2D) but lower insulin responses (0.70 ± 0.073 vs 0.49 ± 0.072; Cre^−^ vs Cre^+^; *P* < .05; 30-min time point; repeated measures two-way ANOVA; n = 14 Cre^−^ and n = 13 Cre^+^) (Figure 2E) in response to a 3-g/kg bodyweight glucose injection, consistent with impaired insulin secretion or enhanced clearance of the hormone. Insulin sensitivity measured using an insulin tolerance test was unchanged in both male (Figure 2F) and female (Supplemental Figure 2) Ins1Cre^+/−^:ZnT8^fl/fl^ mice, as assessed at 10 or 8 weeks, respectively. These findings are in line with those in global (14) or mouse insulin 1 promoter-deleted animals (19).

### Unchanged glucose-, incretin-, and KCl-stimulated insulin secretion but altered Zn^2+^ dynamics in isolated Ins1Cre^+/−^:ZnT8^fl/fl^ islets

**Figure 3.**
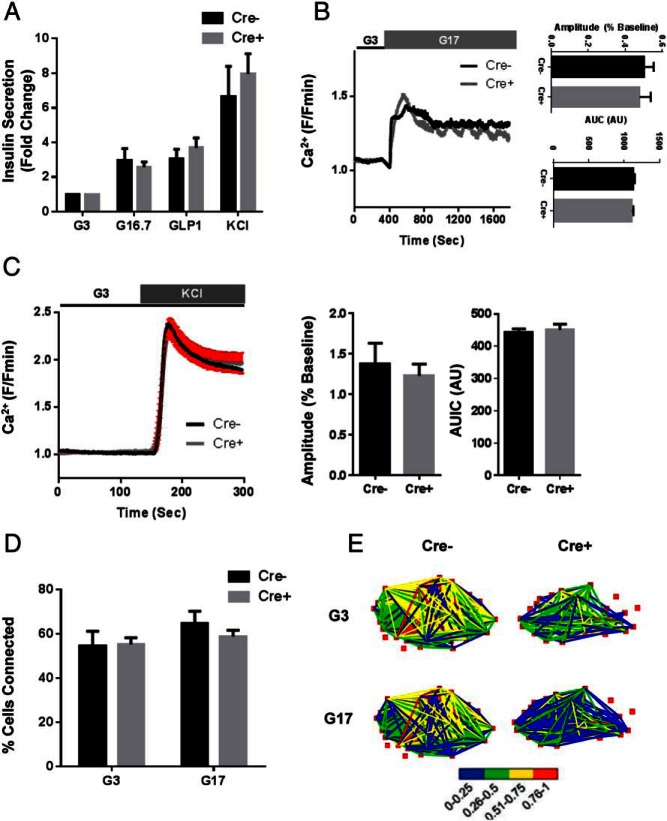
In vitro assessment of islets isolated from Ins1CreZnT8KO mice. Insulin secretion from 5 size-matched islets was assessed using a Homogenous Time-Resolved Fluorescence-based assay (Materials and Methods). Briefly, islets were pretreated at 3mM glucose for 1 hour at 37°C before being exposed to either 3mM glucose (G3), 16.7mM glucose (G16.7), 16.7mM glucose plus 20nM GLP-1 (GLP-1), or 3mM glucose + 30mM KCl (KCl) for 30 minutes at 37°C. Secreted insulin was determined and, after normalization to total insulin, expressed as fold change vs the 3mM glucose condition (A). [Ca^2+^]_i_ dynamics were assessed using Nipkow spinning disk microscopy. No significant differences were seen in either the amplitude or the AUC of glucose-evoked (17mM; G17) whole-islet Ca^2+^ rises (ns vs Cre^−^; Student's *t* test) (B). The number of glucose-responsive cells was unchanged between Cre^−^ and Cre^+^ mice (data not shown). Likewise, there were no differences seen in KCl-induced (30mM KCl) [Ca^2+^]_i_ rises (ns vs Cre^−^; Student's *t* test) (C). Correlation analyses of glucose-evoked Ca^2+^ traces (32) showed no difference in β-cell-β-cell connectivity in Cre^+^ islets vs Cre^−^ islets at both low (3mM) and high (11mM) glucose, indicating maintained cell quiescence and synchronicity, respectively. D, Connectivity map depicting location, number, and strength (color coded; 0 [blue] = lowest, 1 [red] = highest) of significantly correlated cell pairs (E). Values represent mean ± SEM.

Islets isolated from 10-week-old male Ins1Cre^+/−^:ZnT8^fl/fl^ mice showed no change with respect to control islets in in vitro insulin secretion in response to 16.7 mmol/L glucose, incretin or depolarization induced with KCl (ns; two-way ANOVA; n = 12–16 replicates per genotype) (Figure 3A). Secretion in response to lower (8 mmol/L) glucose concentrations was also unchanged (Supplemental Figure 3A) and, similarly, glucose-stimulated insulin release was not different between null and wild-type islets assayed during perifusion at 16.7 mmol/L glucose (Supplemental Figure 3B). Likewise, deletion of ZnT8 did not affect the amplitude (0.50 ± 0.05 vs 0.47 ± 0.06; Cre^−^ vs Cre^+^; ns; Student's *t* test; n = 17 and 9 islets, respectively) or area under the curve (AUC) (1132 ± 19.7 vs 1107 ± 24.2; Cre^−^ vs Cre^+^; ns; Student's *t* test; n = 17 and 9 islets, respectively) of glucose (Figure 3B) or KCl-stimulated intracellular free Ca^2+^ ([Ca^2+^]_i_) increases (amplitude, 1.38 ± 0.11 vs 1.23 ± 0.15; AUC, 443 ± 9.71 vs 450 ± 17.;3 Cre^−^ vs Cre+; ns; Student's *t* test; n = 5 and n = 13 islets, respectively) (Figure 3C). Finally, β-cell-β-cell connectivity (31), known to contribute to the regulation of insulin release from intact islets, was unaltered in ZnT8 null islets (Figure 3, D and E).

**Figure 4.**
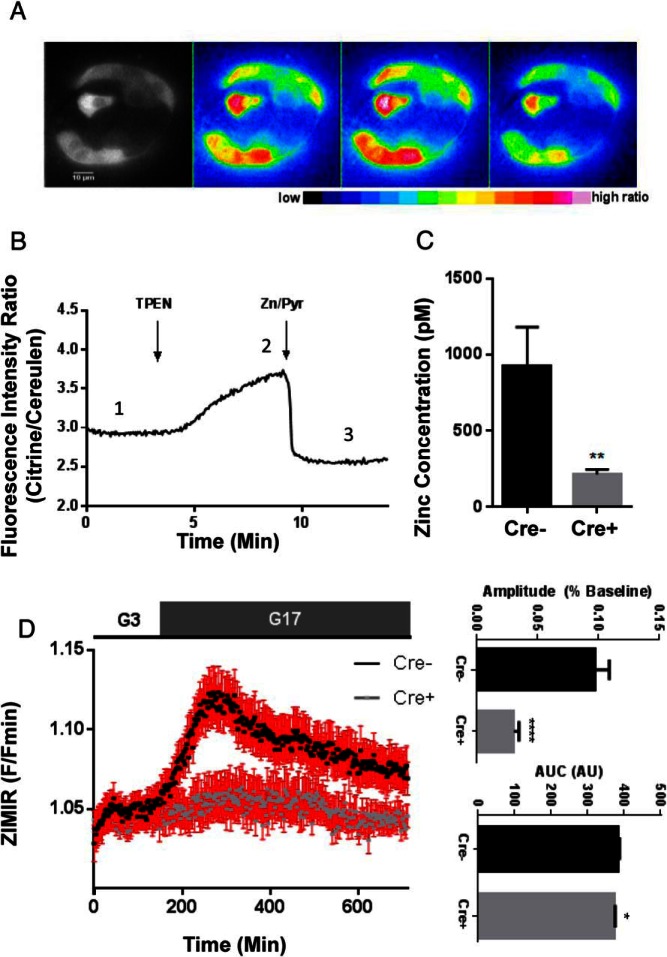
Intracellular zinc dynamics and secretion in control in Ins1CreZnT8KO mouse β-cells. To measure cytosolic free Zn^2+^ levels, isolated islets were dispersed and infected with an adenovirus expressing the Zn^2+^-sensitive FRET probe eCALWY4 (34) (A). Steady-state fluorescence intensity ratio (citrine to cerulean) was first measured 1) before obtaining the Rmax, 2) under perifusion with Krebs-Henseleit Buffer buffer containing the zinc chelator N,N,N′,N′-Tetrakis(2-pyridylmethyl)ethylenediamine (50μM; zinc-free condition). Finally, 3) the Rmin was obtained under perfusion with Krebs-Henseleit Buffer buffer containing 5μM pyrithione and 100μM Zn^2+^ (zinc-saturated condition), providing saturating intracellular Zn^2+^ concentrations (B). The free cytosolic concentration of Zn^2+^ (C) was calculated using the next formula: [Zn^2+^] = Kd (Rmax − R)/(R − Rmin), revealing significant decreases in cytosolic free zinc levels in Ins1Cre:ZnT8^fl/fl^ animals compared with littermate controls (**, *P* < .01, Cre^+^ vs Cre^−^, respectively, n = 20 Cre^+^ and n = 11 Cre^−^ islets). Zinc secretion from isolated islets, using the zinc binding probe zinc indicator for monitoring induced exocytotic release (ZIMIR), was decreased in Cre^+^ islets as shown by significant decreases in both the amplitude (****, *P* < .0001, Student's *t* test) and AUC (*, *P* < .05, Student's *t* test) of glucose-evoked ZIMIR responses; n = 22 Cre^−^ islets and n = 17 Cre^+^ islets (D).

We next used the recombinant Förster resonance energy transfer (FRET)-based probe eCALWY4 (32, 33) to measure cytosolic Zn^2+^ concentrations. Consistent with findings in global ZnT8 null mice (34), Ins1Cre^+/−^:ZnT8^f/lfl^ β-cells showed a significant reduction in free cytosolic Zn^2+^ concentration (920 0.3 ± 261pM vs 212.8 ± 32.5pM; **, *P* < .01, Cre^−^ vs Cre^+^, Student's *t* test, n = 20 and 11 islets, respectively) (Figure 4, A–C). Furthermore, use of the cell surface-targeted Zn^2+^ binding probe zinc indicator for monitoring induced exocytotic release (ZIMIR) (35), to measure Zn^2+^ cosecreted from insulin granules, demonstrated that Ins1Cre^+/−^:ZnT8^fl/fl^ islets secreted substantially less Zn^2+^ compared with control islets after stimulation with glucose (Figure 4D).

### Ins1Cre^+/−^:ZnT8^f/f^ mice show altered insulin granule morphology but preserved β-cell mass

**Figure 5.**
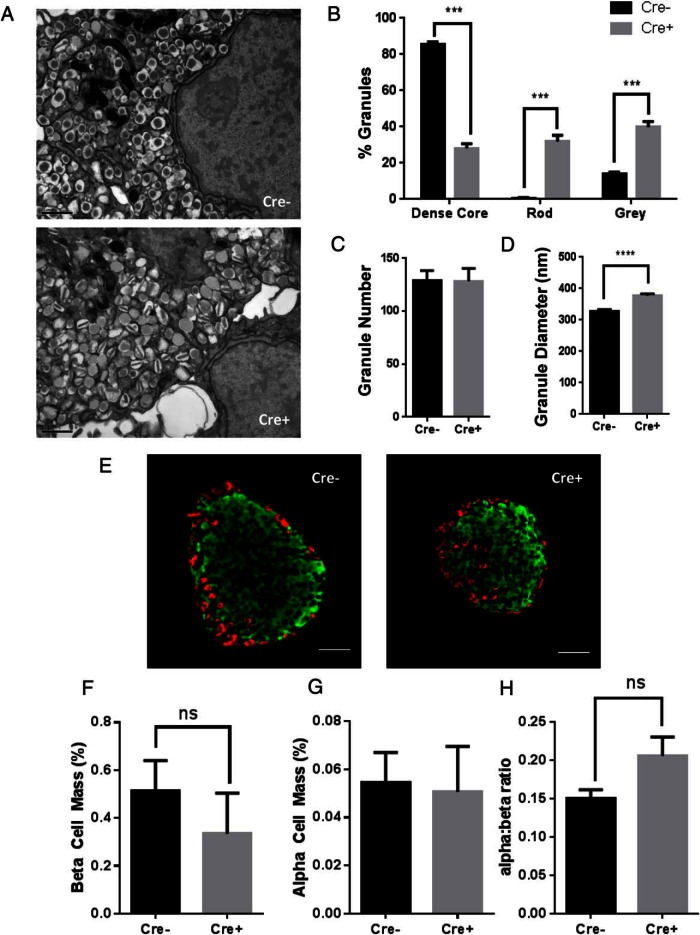
Ins1CreZnT8KO mice exhibit abnormal granule morphology but unchanged β-cell mass. Transmission electron microscopic images of β-cells from islets isolated from Cre^−^ (upper) or Cre^+^ (lower) animals show altered dense core granule structure. Scale bar, 1 μm. A, Insulin granules were grouped into 3 categories according to morphological structure and counted. Cre^+^ animals showed a significant reduction in granules containing a dense core coupled with significant increases in granules showing a rod like structure or a gray interior (***, <*P* < .001 vs Cre^−^) (B). No changes in total granule number were seen between genotypes (ns vs Cre^−^) (C). Granule diameter was increased in Cre^+^ β-cells (****, *P* < .0001 vs Cre^−^, Student's *t* test) (D). Staining pancreatic slices from Cre^−^ (E, left) and Cre^+^ (E, right) (scale bar, 50 μm) for insulin and glucagon revealed no significant differences in α- to β-cell ratio (F), β-cell mass (G), or α-cell mass (H) (n = 3 animals per genotype). Values represent mean ± SEM.

The above metabolic and cellular disturbances were accompanied by a drastic change in secretory granule morphology (Figure 5A), with a near-complete loss of dense core granules in islets from Ins1Cre^+/−^:ZnT8^fl/fl^ mice, and the emergence of “atypical” granules possessing abnormal “rod-like” (0.65 ± 0.28% vs 31.9 ± 3.42%, Cre^−^ vs Cre^+^, *P* < .0001, n = 8 β-cells/genotype) or “empty” (36.3 ± 2.48% vs 85.6 ± 1.37%, Cre^−^ vs Cre^+^, *P* < .0001, n = 8 β-cells/genotype) cores (Figure 5B). The total number of granules was unchanged (129 ± 9.25 vs 128 ± 11.9; Cre^−^ vs Cre^+^; ns; Student's *t* test; n = 8 β-cells/genotype) (Figure 5C), but average granule diameter was increased (377.47 ± 5.12 vs 328.09 ± 5.46 nm, Cre^+^ vs Cre^−^, *P* < .001, n = 204/226 granules) (Figure 5D), likely reflecting increased osmotic stress resulting from free electrolytes in the ZnT8 null granule (18). Staining pancreatic slices for insulin and glucagon (Figure 5E) showed no changes in total β-cell mass (0.513 ± 0.07% vs 0.334 ± 0.17%; Cre^−^ vs Cre^+^; ns; Student's *t* test; n = 3 animals per genotype) (Figure 5F), α-cell mass (0.055 ± 0.015% vs 0.051 ± 0.019%; Cre^−^ vs Cre^+^; ns; Student's *t* test; n = 3 animals per genotype) (Figure 5G) or α- to β-cell ratio (0.151 ± 0.011 vs 0.206 ± 0.025; Cre^−^ vs Cre^+^; ns; Student's *t* test; n = 3 animals per genotype) (Figure 5H). These data are in line with previous findings using alternative deleter strains to eliminate ZnT8 from the β-cell (14, 18, 36).

The above studies thus demonstrate that deleting ZnT8 selectively in the β-cell leads to normal insulin, but abnormal Zn^2+^ secretion, in vitro, but markedly lower poststimulation insulin levels and glucose tolerance in vivo.

### Improved glucose tolerance in ZnT8 transgenic mice

**Figure 6.**
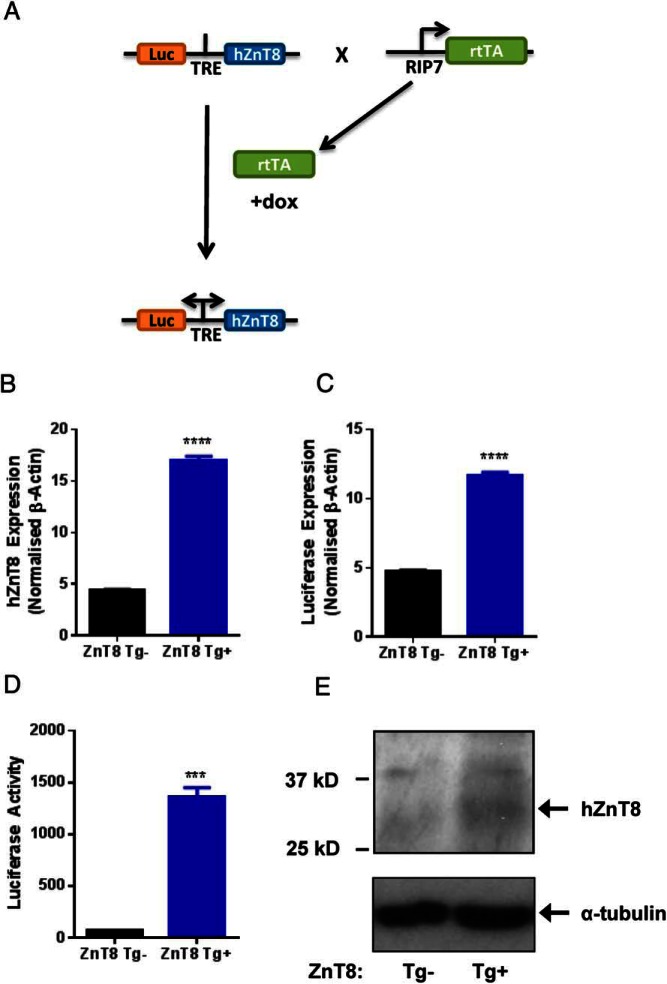
Selective overexpression of ZnT8 in the mouse β-cell. Mice expressing human ZnT8 (hZnT8) and luciferase under the control of a bidirectional, tetracycline-regulated promoter were crossed with *RIP7-rtTA* mice (28) and bred to give ZnT8 Tg^+^ mice (*Rip7-rtTA*^+^*ZnT8Tg*^+^) and littermate control animals (*Rip7-rtTA*^+^*ZnT8*^−^). Administration of doxycycline in the drinking water was used to induce ZnT8 overexpression specifically in the β-cell (A). Quantitative PCR (qPCR) revealed increased hZnT8 (****, *P* < .0001 vs ZnT8 Tg^−^, n = 3 animals per genotype) (B) and luciferase (****, *P* < .0001 vs ZnT8 Tg^−^, n = 3 animals per genotype) (C) gene expression in isolated mouse islets. Luciferase activity was also increased in islets isolated from ZnT8 Tg^+^ animals (***, *P* < .001 vs ZnT8 Tg^−^, n = 4) (D). Western (immuno)blotting demonstrated increased hZnT8 protein expression in isolated islets from transgenic mice (E).Values represent mean ± SEM.

To explore the impact of increasing ZnT8 activity in β-cells we next generated transgenic mice in which the expression of the transporter was under the control of a bidirectional tetracycline-inducible promoter (37). β-Cell-selective induction was then achieved by activation of a Tet-On transactivator expressed selectively in β-cells under the control of the rat insulin 2 promoter (RIP7-rtTA) (Figure 6A). Although 7 founders were produced, we selected 2 (31 and 23; copy numbers 5 and 13, respectively) for further analysis (Supplemental Figure 4). Treatment with doxycycline of transgenic animals derived from founder 31 (5 copies) resulted in an approximately 4-fold induction of human ZnT8 mRNA expression (4.40 ± 0.08 vs 16.9 ± 0.40; ZnT8 Tg^−^ vs ZnT8 Tg^+^; *P* < .0001; Student's *t* test; n = 3 per genotype) (Figure 6B). This was accompanied by both an increase in luciferase mRNA expression (4.78 ± 0.06 vs 11.7 ± 0.25; ZnT8 Tg^−^ vs ZnT8 Tg^+^; *P* < .0001; Student's *t* test; n = 3 each genotype) (Figure 6C) and activity in isolated islets (68.33 ± 2.85 vs 1362 ± 86.9; ZnT8 Tg^−^ vs ZnT8 Tg^+^; *P* < .0001; Student's *t* test; n = 3 each genotype) (Figure 6D). The expression of human ZnT8 protein was also apparent by Western blotting using an antibody specific for the human protein (Figure 6E).

**Figure 7.**
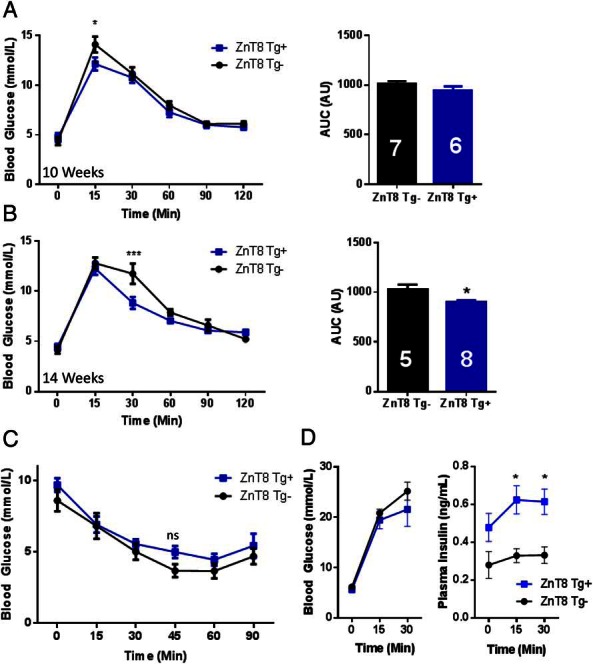
Female ZnT8 Tg^+^ mice show improvements in glucose tolerance. Glucose tolerance of Rip7-rtTA^+^ZnT8Tg^+^ animals was assessed by ip glucose tolerance test (IPGTT). A significant improvement in glucose tolerance was seen in 10-week-old (*, *P* < .05, 15-min time point) (A) and 14-week-old (**, *P* < .01, 30-min time point; *, *P* < .05 AUC) (B) female animals. Insulin sensitivity was unchanged by ZnT8 overexpression (C). Plasma glucose (D, left) tended to be reduced in ZnT8 Tg^+^ mice, but insulin (D, right) levels were significantly increased in response to a 3-g/kg bodyweight ip injection of glucose (*, *P* < .05, 15- and 30-min time points). Values represent mean ± SEM.

Females from founder 31 displayed significant improvements in glucose tolerance at both 10 weeks of age (12.2 ± 0.51 vs 13.9 ± 1.1 mmol/L; ZnT8 Tg^+^ vs ZnT8 Tg^−^; *P* < .05, 15-min time point; repeated measures two-way ANOVA; n = 6 and 7, respectively) (Figure 7A) and 14 weeks of age (8.84 ± 0.59 vs 11.7 ± 1.01 mmol/L; ZnT8 Tg^+^ vs ZnT8 Tg^−^; *P* < .001, 30-min time point, repeated measures two-way ANOVA; *, *P* < .05, AUC, Student's *t* test, n = 8 and 5, respectively) (Figure 7B), whereas changes were not apparent in males (Supplemental Figure 5). Insulin sensitivity was unchanged in 10-week-old female ZnT8 transgene positive animals compared with wild-type littermates (ns; repeated measures two-way ANOVA; n = 4 ZnT8 Tg^−^ and 5 ZnT8 Tg^+^) (Figure 7C). Measured in vivo after ip injection of 3-g/kg glucose, insulin secretion was significantly enhanced by almost 2-fold compared with wild-type littermates, despite a tendency towards lowered blood glucose levels (*, *P* < .05; 15- and 30-min time point; repeated measures two-way ANOVA; n = 6 ZnT8 Tg^−^ and n = 8 ZnT8 Tg^+^ animals) (Figure 7D).

Both male and female mice derived from founder 23 (copy number 13) displayed no apparent changes in glucose tolerance at either 10 or 14 weeks of age (Supplemental Figure 6, A–D), consistent with the significantly lower levels of overexpression of the transgene and coexpressed reporter gene (luciferase) in this line vs line 31 (Supplemental Figure 4).

### ZnT8 Tg^+^ islets secrete less insulin but more Zn^2+^ in response to glucose

**Figure 8.**
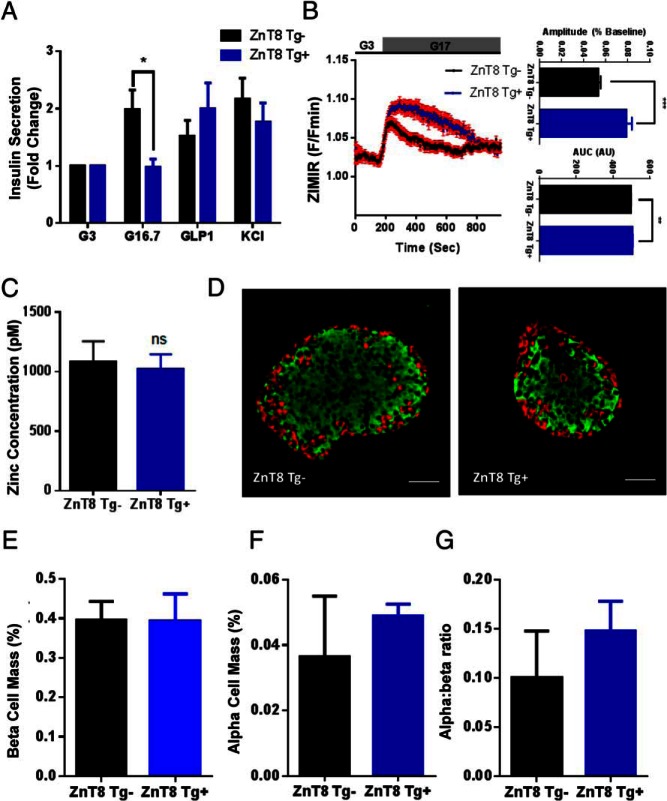
Insulin secretion and endocrine cell mass in ZnT8 Tg^+^ islets. Glucose-stimulated insulin secretion was significantly reduced in isolated islets (*, *P* < .05) (A), whereas Zn^2+^ secretion was enhanced as shown by significant increases in both the amplitude (***, *P* < .001) (B, top) and AUC (**, *P* < .01) (B, bottom) of glucose-stimulated ZIMIR responses. Cytosolic Zn^2+^ concentrations, measured using eCALWY4 (Figure 4), were unchanged by ZnT8 overexpression (C). Staining pancreatic slices for insulin and glucagon (scale bar, 50 μm) (D) revealed no changes in β-cell (E) or α-cell (F) mass nor in α- to β-cell ratio (G). Values represent mean ± SEM.

Assayed in vitro, insulin secretion from isolated islets derived from 10- to 14-week-old female mice in response to high glucose (16.7mM) was significantly reduced (0.94 ± 0.21 vs 0.40 ± 0.05 ng/mL, ZnT8 Tg^−^ vs ZnT8 Tg^+^, respectively; *P* < .05; two-way ANOVA, n = 10–13 replicates) (Figure 8A). No differences were apparent in the response of transgenic islets to stimulation with incretin or depolarization with KCl (Figure 8A).

Demonstrating enhanced glucose-stimulated Zn^2+^ secretion from transgenic mouse islets, both the amplitude (0.053 ± 0.002 vs 0.791 ± 0.05; ZnT8 Tg^−^ vs ZnT8 Tg^+^, respectively; *P* < .001; two-way ANOVA, n = 11 and 14, respectively) and AUC (496 ± 1.72 vs 505 ± 2.11; ZnT8 Tg^−^ vs ZnT8 Tg^+^, respectively; *P* < .01; n = 11 and 14, respectively) of glucose-evoked ZIMIR responses (Figure 8B) were increased by ZnT8 overexpression. Interestingly, there were no changes in cytosolic Zn^2+^ concentrations (1079 ± 176pM vs 1020 ± 127pM; ZnT8 Tg^−^ vs ZnT8 Tg^+^; ns; Student's *t* test; n = 46 and 61 cells, respectively, from 2–6 animals per genotype) (Figure 8C).

Staining pancreatic slices for insulin and glucagon (Figure 8D) revealed no changes in β- or α-cell mass nor the ratio of β- to α-cells (Figure 8, E–G).

## Discussion

In this report, we describe new mouse models for ZnT8, which provide insights into the pathogenic mechanisms likely to be involved in the actions of human alleles associated with increased T2D risk.

Ins1CreZnT8KO mice showed dramatic changes in secretory granule morphology and plasma insulin level under glucose stimulation, similar to findings previously reported in global ZnT8 KO mice (14), in mice with conditional ZnT8 alleles deleted with the more promiscuous RIP2Cre (15), or with MIPCre which also expresses GH (24). The present results thus confirm that such morphological changes are likely to be a β-cell-autonomous event and to reflect impaired Zn^2+^ uptake into dense core granules in the absence of ZnT8.

Despite the exaggerated glucose excursions and smaller plasma insulin increases observed in response to ip injection of the sugar in these animals (Figure 2, B and E, respectively), islets derived from Ins1CreZnT8KO mice displayed unaltered glucose-stimulated insulin secretion in vitro. By contrast, glucose-induced Zn^2+^ release from these islets was reduced by more than 80%, in line with earlier results with global ZnT8 KO mice (36), presumably reflecting impaired Zn^2+^ accumulation by secretory granules. These findings reinforce the recent proposal (19, 34) that impaired β-cell Zn^2+^ secretion and deinhibition of insulin receptor endocytosis leads to exaggerated clearance of mature insulin by the liver.

We extend support for the above view by showing that glucose tolerance is improved in a new model in which ZnT8 is selectively overexpressed in the β-cell. Remarkably, insulin secretion from islets isolated from these mice was barely stimulated by glucose, whereas Zn^2+^ release was increased by more than 50%. Nonetheless, fasting insulin levels tended to be increased in ZnT8Tg animals (Figure 7D), and these levels were further strongly increased by ip glucose injection (Figure 7D). Thus, in ZnT8Tg animals, elevated Zn^2+^ secretion may act both to impair insulin clearance through the internalization of insulin receptors (19), and possibly also to enhance insulin signaling. At the molecular level, possible actions of the released Zn^2+^ included inhibition of insulin receptor dephosphorylation by protein tyrosine phosphatase B1 (38), or of phosphatidylinositol (3,4,5) phosphate degradation by phosphatase and tensin homologue on chromosome 10 (39).

**Figure 9.**
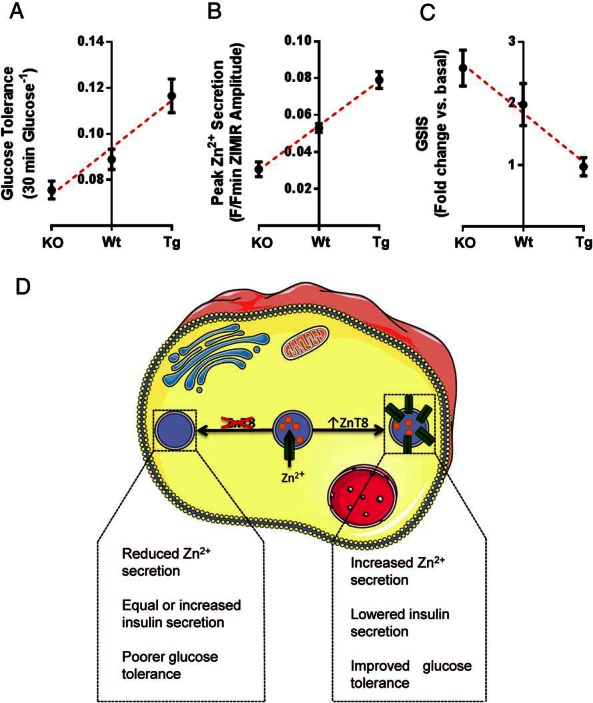
Impact of ZnT8 manipulation in pancreatic β-cells on murine glucose homeostasis. Although glucose tolerance (A) and Zn^2+^ secretion (B) are both increased with increasing ZnT8 levels, glucose-stimulated insulin secretion is impaired (C). Data are taken from Figures 2–4, 7, and 8. Schematic representation, deletion of ZnT8 specifically in pancreatic β-cells leads to impaired glucose tolerance and abnormal insulin granule morphology. Conversely, overexpressing ZnT8 in the β-cell causes improvements in glucose tolerance but reduced glucose-stimulated insulin secretion (D).

These studies show that, by manipulating *Slc30a8* expression selectively in the mouse β-cell using molecular genetics, a near-linear relationship exists in this species between ZnT8 levels and glucose tolerance (Figure 9A). Note that the study of mice deleted for just one conditional *Slc30a8* allele was not feasible with the breeding strategy used here though, in earlier studies with global *Slc30a8* null mice (14), we noted that heterozygous (ZnT8^+/−^) mice displayed intermediate glucose tolerance between wild-type and homozygous null animals, consistent with the current findings. Importantly, the changes in peak glucose observed in the present study were best correlated to Zn^2+^ release from the islet (Figure 9B): this was essentially eliminated by *Slc308* deletion (Figure 4D) but enhanced when the transporter was overexpressed (Figure 8B). By contrast, insulin release in vitro was inversely correlated with ZnT8 expression (Figure 8C).

Interestingly, we observed differences between the impact of *Slc308* deletion on male and female mice, with only males showing defective glucose tolerance over the age range examined. In contrast, both male and female mice deleted globally for the transporter (14) displayed glucose intolerance at 6 weeks of age, whereas only males were intolerant at 12 weeks. The reasons for the differences between the impact of *Slc308* deletion between sexes is presently unknown but may in part reflect the intrinsically greater intermeasurement variability in females resulting from the reproductive cycle, and/or the lower insulin sensitivity of male animals, which imposes a greater metabolic stress on the β-cell. Surprisingly, this position was reversed in transgenic animals with the greater penetrance of ZnT8 overexpression observed in females. In this case, the underlying mechanisms are less clear but might reflect sex-specific differences in the handling of enhanced Zn^2+^ loads by the liver or other target tissues (19).

Whether control of hepatic insulin clearance and/or action via Zn^2+^ assumes the same importance in man, where the much larger diameter and volume of the portal vein may mean greater dilution of Zn^2+^ after release from the β-cell, and hence lowered action on the liver, is unclear. Nonetheless, and arguing for this possibility, carriers of risk (R) *SLC30A8* alleles show lowered C-peptide:insulin levels, consistent with the more efficient uptake of the latter by hepatocytes when Zn^2+^ levels are lowered (40).

The present approach further demonstrates the feasibility of using mouse genetics to explore the mechanisms through which T2D risk genes, identified in GWAS studies (12), act. Intriguingly, we provide additional evidence that the actions of *SLC30A8* involve interactions between multiple tissues (β-cells and liver), despite the tight restriction of the expression of this gene to the endocrine pancreas (13). Whether *SLC30A8* variants also influence the release of glucagon may require further investigation; global inactivation of the gene exerted little effect on glucagon release from islets, although detailed in vivo analysis involving hypoglycemic clamps were not reported in these studies (15). Fadista et al (41) recently reported a strong positive correlation between glucagon and *SLC30A8* expression in human islets, consistent with a role for *SLC30A8* variants in controlling glucagon production.

In the light of the present results, the possibility that other GWAS genes expressed in multiple tissues, eg, *TCF7L2* (42, 43), might act via extrapancreatic sites to regulate insulin secretion, would seem worthy of careful investigation. Of note, TCF7L2 is an upstream regulator of the mouse *Slc30a8* (44) and human *SLC30A* (45) genes and, as a “master” regulator of T2D susceptibility (45), might act in part via ZnT8 to modify β-cell Zn^2+^ release and insulin clearance.

## Materials and Methods

### Ethical approval

All animal procedures were approved by the home office according to the Animals (Scientific Procedures) Act 1986 of the United Kingdom (PPL 70/7349).

### Generation of β-cell-selective knockout mice by Ins1Cre-driven recombination

ZnT8 floxed mice (ZnT8^fl/fl^) were generated by GenOway (15). This involved the insertion of a LoxP site together with a flippase recognition target flanked neomycin selection cassette within intron 1 and a single distal LoxP site within the upstream exon 1 containing the translational start codon. ZnT8^fl/fl^ animals were then bred with the Ins1Cre deleter strain, to produce 50% β-cell-specific knockout animals (Ins1Cre^+/−^ZnT8^fl/fl^) and 50% littermate controls (Ins1 Cre^−/−^ZnT8^fl/fl^). Note that, in contrast to RIP2Cre (24) and MIP2Cre (19), Ins1Cre mice do not express a GH cassette, and the transgene alone does not affect glucose tolerance (25, 29). Animals were maintained in a pathogen-free facility under a 12-hour light, 12-hour dark cycle with free access to water and food.

### Generation of β-cell-specific transgenic mice

Plasmid pCDNA3, containing the human *ZnT8* (W325 form) coding sequence with the addition of a single COOH-terminal c-*Myc* epitope tag (14), was digested with *Xho*I, blunt-end filled, and further digested with *Not*I. The digested *hZnT8-Myc* DNA fragment was gel purified and cloned into plasmid pBI-L Tet (Clontech) between *Not*I and *Pvu*II sites. This generated a plasmid with a bidirectional tetracycline-regulated promoter driving expression of both *hZnT8-Myc* and firefly luciferase. The positive clone was further confirmed by DNA sequencing using a pBI-L internal primer GAAAGAACAATCAAGGGTCC and a *hZnT8* primer ACACTAGCACGCCAGTCACC.

The expression cassette was excised from the plasmid backbone by *Aat*II and *Ase*I digestion and transferred by pronuclear microinjection into C57Bl/6 mouse oocytes (MRC Clinical Sciences Centre transgenic facility, Hammersmith Hospital, Imperial College London). Successful integration was identified by PCR screening of DNA extracted from ear biopsies by the HotSHOT method (46) using 2 sets of primers: 1) *hZnT8* gene forward, CTGTCATCGAAGCCTCCCTC and reverse, AAGGGCATGCACAAAAGCAG; and 2) Luciferase gene forward, CATTAAAACCGGGAGGTAGATGA and reverse, CATGGATTCTAAAACGGATTACCA. The relative transgene copy number was determined by SYBR green quantitative PCR method (Life Technology) using a set of luciferase gene primers: forward, CAACTGCATAAGGCTATGAAGAGA and reverse, ATTTGTATTCAGCCCATATCGTTT, and, as an internal control, a set of mouse *Cxcl12* gene primers: forward, GGACGAGCTCCACTTAGACG and reverse, CAACATGTCCAGATC GAAATC. Two founders were crossed twice with C57Bl/6 mice to generate the *hZnT8-Luc* strain. *RIP7-rtTA* mice on a C57Bl/6 background (28), expressing the reverse tetracycline transactivator under the control of the rat insulin promoter, were crossed with *hZnT8-Luc* mice to permit β-cell-specific, tetracycline-inducible expression of *hZnT8-Myc* and luciferase. *hZnT8-Luc* mice were crossed with homozygous *RIP7-rtTA* mice to produce littermates of 2 genotypes as follows: *hZnT8-Luc*^+^/*RIP7-rtTA*^+^ (ZnT8 Tg^+^) and *hZnT8*^−^*Luc*^−^*/RIP7-rtTA*^+^ (ZnT8 Tg^−^). All offspring were genotyped for both the *hZnT8* and *RIP7-rtTA* genes (47). Mice were treated with 0.5-g/L doxycycline from 5 weeks of age.

### Islet isolation

Mice were euthanized by cervical dislocation and pancreatic islets isolated by collagenase digestion as preciously described (48). Given the sex- and age-dependent differences between mouse lines, islets used for ex vivo analysis were obtained from mice of the appropriate sex and, importantly, at an age where an in vivo phenotype was apparent ie, 10-week-old male Ins1Cre^+/−^::ZnT8^fl/fl^ mice and 10- to 14-week-old female Rip7rTta^+/−^::ZnT8Tg^+/−^ mice.

### Quantitative real-time PCR

Total islet RNA was extracted using TRIzol reagent (Invitrogen). After reverse transcription, relative expression was assessed using SYBR Green (Invitrogen). Primers were designed using PerlPrimer and gene expression was normalized to β-actin (*Actb*).

### Immunofluorescence

Isolated islets were fixed overnight at 4°C in 4% paraformaldehyde (vol/vol) before the addition of primary antibodies against murine ZnT8 (1:200, raised in rabbit; Mellitech) insulin (1:200, raised in guinea pig; DAKO), and glucagon (1:1000 raised in mouse; Sigma-Aldrich). Detection was performed using goat antirabbit Alexa Fluor 488, goat antiguinea pig Alexa Fluor 568, and goat antimouse Alexa Fluor 568 (1:500; all Invitrogen). Islets were mounted on Superfrost slides (Fisher Scientific) using Vectashield 4′,6-diamidino-2-phenylindole-containing hardset mounting medium (Vector Laboratories). Data capture was performed using a Zeiss LSM780 confocal microscope equipped with GaAsP spectral detectors and a ×64/1.4NA oil-immersion objective. The proportion of ZnT8-immunopositive α- and β-cells was quantified according to colocalization of ZnT8 with either insulin or glucagon, above a background threshold (ie, twice the signal to noise ratio). In all cases, uniform linear adjustments were applied to contrast/brightness to improve image quality for presentation while preserving the pixel dynamic range. Background fluorescence after insulin staining is likely to correspond to autofluorescence and was left uncorrected to preserve image integrity for comparisons. Likewise, stippled background fluorescence after glucagon staining can be attributed to nonspecific staining apparent when the ZnT8 antibody was used in the presence of the antiglucagon antibody.

### Histology and immunohistochemistry

Mouse pancreata were extracted and fixed in 10% neutral balanced formalin (Sigma) at 4°C for 18 hours before dehydration and wax embedding and processing to obtain 5-μm slices. Sections were labeled with antiinsulin (1:200 dilution; secondary Alexa Fluor 488, 1:1000) and antiglucagon (1:100 dilution; secondary Alexa Fluor 568, 1:500) and sealed using Vector Shield Antifade Hard Set reagent (Vector Laboratories). β-Cell mass was determined as described (49). Data capture was performed using a Zeiss AxioObserver and a ×40/0.75NA objective. β/α-Cell mass was calculated using the threshold plugin for ImageJ (NIH), as previously detailed (50).

### Intraperitoneal glucose and insulin tolerance tests

Glucose (1-g/kg bodyweight) was injected into the abdomen of mice that had been fasted overnight. Blood glucose measurements were taken at 0, 15, 30, 60, 90, and 120 minutes using an automatic glucometer (Accucheck). Insulin tolerance tests are performed as per glucose tolerance test but animals were fasted for 5 hours before 0.75-U insulin/kg bodyweight insulin injection.

### Plasma insulin measurements

Mice fasted overnight were injected with glucose (3-g glucose/kg bodyweight) and blood from the tail vein was collected into heparin coated tubes (Sarstedt) at 0, 15, and 30 minutes. Plasma was separated by centrifugation at 2000*g* for 10 minutes, 5 μL of blood plasma were used to measure insulin levels using an ultrasensitive mouse insulin ELISA kit (Crystal Chem).

### Insulin secretion assay

Five size matched islets were pre incubated for 1 hour at 37°C in a Krebs-HEPES-bicarbonate (KHB) buffer (130mM NaCl, 3.6mM KCl, 1.5mM CaCl_2_, 0.5mM MgSO_4_, 0.5mM NaH_2_PO_4_, 2mM NaHCO_3_, 10mM HEPES, and 0.1% [wt/vol] BSA; pH 7.4) containing 3 mmol/L glucose with gentle shaking (120 rpm). Islets were further incubated for 30 minutes at 37°C in either: 3 mmol/L glucose, 8 mmol/L glucose, 16.7 mmol/L glucose, 16.7 mmol/L glucose plus 20 nmol/L GLP1, or 3 mmol/L glucose plus 30 mmol/L KCl, before collection of supernatant fractions for insulin secretion analysis. Total insulin was collected by lysing islets in 500 μL acidified ethanol solution (1.5% [vol/vol] HCl, 75% [vol/vol] ethanol, and 0.1% [vol/vol] Triton X-100) followed by sonication. Secreted and total insulin was measured using Homogenous Time-Resolved Fluorescence assay kit (Cisbio). Insulin release during perifusion was monitored using a custom-built device. Experiments were performed in triplicate and 50 islets were perifused at a rate of 500 μL min^−1^ at 37°C.

### [Ca^2+^]_i_ imaging and connectivity analysis

Isolated islets were incubated (37°C, 95% O_2_/5% CO_2_) for 1 hour in fluo 2-AM (10μM; Teflabs) diluted in a HEPES-bicarbonate buffer solution (120mM NaCl, 4.8mM KCl, 1.25mM NaH_2_PO_4_, 24mM NaHCO_3_, 2.5mM CaCl_2_, 1.2mM MgCl_2_, 10mM HEPES, and 3mM D-glucose; all Sigma). Functional multicellular Ca^2+^ imaging was achieved using a Nipkow spinning disk head allowing rapid scanning of islet areas for long periods of time with minimal phototoxicity. A solid-state laser (CrystaLaser) controlled by a laser-merge module (Spectral Applied Physics) provided wavelengths of 491 nm to excite fluo-2 (rate, 0.5 Hz; exposure time, 600 ms). Emitted light was filtered at 525/50 nm, and images were captured by a highly sensitive 16-bit, 512 × 512 pixel back-illuminated EM-CCD camera (ImageEM 9100–13; Hamamatsu). Volocity software (PerkinElmer) provided the user interface. During recordings, islets were maintained at 35°C–36°C and continuously irrigated with bicarbonate buffer aerated with 95% O_2_/5% CO_2_. Connectivity analysis was performed as previously described (31).

### ZIMIR imaging

ZIMIR imaging was performed as previously described (36). Briefly, isolated islets were incubated (37°C, 95% O_2_/5% CO_2_) in ZIMIR (1μM) for 30 minutes and imaged in bicarbonate buffer solution supplemented with 1μM EDTA to improve the signal to noise ratio. ZIMIR was excited at 491 nm and emitted signals captured at 525 nm. After acquisition, islets were divided into subregions before extraction of intensity over time to allow analysis of amplitude and AUC of glucose-stimulated ZIMIR responses.

### Cytosolic free Zn^2+^ measurements

Zn^2+^ measurements were acquired as previously described (31). Briefly, islets were dispersed onto coverslips before infection with adenovirus containing the FRET-based Zn^2+^ sensor eCALWY4. Steady-state fluorescence intensity ratio citrine to cerulean (R) was measured, then maximum ratio (Rmax) and minimum ratio (Rmin) were determined to calculate free Zn^2+^ concentration using the next formula: [Zn^2+^] = Kd (Rmax − R)/(R − Rmin). The Rmax was obtained upon intracellular zinc chelation with 50μM N,N,N′,N′-Tetrakis(2-pyridylmethyl)ethylenediamine and the Rmin was obtain upon Zn^2+^ saturation with 100μM ZnCl2 in the presence of the Zn^2+^ ionophore, pyrithione (5μM).

### Protein extraction and Western (immuno)blotting analysis

For protein analysis, roughly 100 islets were washed twice in ice-cold PBS and lysed in ice-cold radioimmunoprecipitation assay buffer (50mM Tris HCl [pH 8.0], 150mM NaCl, 1% nonylphenoxypolyethoxylethanol, 0.5% sodium deoxycholate, and 0.1% sodium dodecyl sulfate). Protein was assayed with a BCA kit (Pierce). Total protein extracts (30 μg) were resolved by sodium dodecyl sulfate-PAGE (8% vol/vol acrylamide) and transferred to polyvinylidene fluoride membranes, followed by immunoblotting with either rabbit polyclonal antimouse or antihuman ZnT8 (both 1:200, Millitech clone R/PZ8) and mouse monoclonal antitubulin (1:5000, Sigma clone B-5–1-2) antibodies. Secondary horse radish peroxidase-linked antirabbit or antimouse antibodies (1:3000; GE Healthcare) were revealed by using enhanced chemiluminescence detection reagent (GE Healthcare).

### Transmission electron microscopy

Isolated islets were fixed in Vincenzo's fixative (2% paraformaldehyde, 2.5% glutaraldehyde, 3mM CaCl_2_, and 0.1M sodium cacodylate buffer [pH 7.4]) for 20 minutes at 37°C initially followed by a further 2 hours at room temperature and finally overnight at 4°C. Electron microscopy was performed as previously described (51).

### Statistical analysis

Values represented are the mean ± SEM. Statistical significance was assessed using either Student's *t* test or the Mann-Whitney *U* test depending on data distributions. Two-way ANOVA (with Bonferroni or Sidak multiple comparison test) was used to examine the effect of multiple variables. Statistical analyses were performed using GraphPad Prism 6.0, ImageJ, and IgorPro.
